# Surgical Suggestions

**Published:** 1906-08

**Authors:** 


SURGICAL SUGGESTIONS. Practical Brevities in Surgical Diagnosis and Treatment. By Walter M. Brickner, M.D., Chief of Surgical Department, Mount Sinai Hospital Dispensary, New York; Editor *American Journal of Surgery,* and Eli Moschcowitz, M.D., Assistant Physician, Mount Sinai Hospital Dispensary, New York; Editorial Associate *American Journal of Surgery.* Duodecimo; 60 pages. New York: Surgery Publishing Co., 1906. Cloth, 50 cents.

This little book is most novel, not only on account of the many original terse and epigrammatic practical suggestions given, but its general appearance and attractive form. It contains 250 suggestions grouped under proper headings and its contents are carefully indexed. While some of the items are familiar to the practical surgeon, they are presented in a manner that will impress them on the reader's memory. The book is bound in heavy cloth, stamped in gold, and the text printed upon India tint paper with marginal headings in red. This book will be much appreciated by the general practitioner, not alone on account of the value of its contents, but as an artistic bit of book making.

